# New Arctic Bacterial Isolates with Relevant Enzymatic Potential

**DOI:** 10.3390/molecules25173930

**Published:** 2020-08-28

**Authors:** Michał Piegza, Wojciech Łaba, Miroslava Kačániová

**Affiliations:** 1 Department of Biotechnology and Food Microbiology, Wrocław University of Environmental and Life Sciences, Chelmonskiego 37, 51-630 Wroclaw, Poland; wojciech.laba@upwr.edu.pl; 2 Department of Fruit Sciences, Viticulture and Enology, Faculty of Horticulture and Landscape Engineering, Slovak University of Agriculture, Tr. A. Hlinku 2, 94976 Nitra, Slovakia; kacaniova.miroslava@gmail.com; 3 Department of Bioenergetics, Food Analysis and Microbiology, Institute of Food Technology and Nutrition, University of Rzeszow, Cwiklinskiej 1, 35-601 Rzeszow, Poland

**Keywords:** MALDI-TOF MS Biotyper, molecular, fluorescence, hydrolytic enzymes

## Abstract

Fragments of wood drifting in the vicinity of Spitzbergen were used for the isolation of microorganisms, carried out using atypical carbon sources: colloidal chitin, cellulose and carboxymethylcellulose, xylan, casein, tributrin and olive oil. Purified cultures were subjected to a three-step identification: with classical methods, using MALDI-TOF MS Biotyper whole-cell protein fingerprinting, and molecular analysis of 16S rDNA. Subsequently, a preliminary assessment of the enzymatic potential of isolates was carried out. As a result, cellulolytic activity was observed in more than 50% of the bacterial strains, exhibiting activity of 0.30–0.40 U/mL. Over 53% of the isolates demonstrated xylanolytic activity, of which the highest reached from 0.40 to 0.90 U. Polygalacturonase activity of 0.003–1.6 was also demonstrated in half of the bacterial strains studied. Proteolytic activity of isolates did not exceed 0.3 U. An important highlight was the ability of fluorescent dye production by certain strains, grown on skim milk-agar, but also on pure meat extract.

## 1. Introduction

An important component of rational environmental management is to maximize the use of already processed resources. One of the elements of this approach is to search for microorganisms located in atypical ecological niches. This allows for obtaining microbial strains with interesting and unique features, e.g., increased resistance to hostile conditions and the capability for the biosynthesis of extracellular hydrolases. 

The expression “ecological niche” was used for the first time by botanist Joseph Grinnell. In 1904, he defined a set of abiotic features of the environment that allows for the growth, development and reproduction of organisms living there. Such features comprise, e.g., temperature, humidity, salinity or oxygen concentration [1]. 

The Arctic, the North Sea region and Spitzbergen have been widely studied and described in terms of the composition of microbial populations. Difficult conditions stimulate microorganisms to develop unusual enzymatic apparata, as well as resistance to environmental factors, which provides a wide range of opportunities for applicatory research in various branches of science. High hopes are associated with organisms isolated from permafrost, which can provide interesting information about the evolutionary processes. An example of such properties is the production of secondary metabolites by *Pseudomonas aeruginosa*, demonstrating antimicrobial activity against *Staphylococcus aureus* or *Candida albicans* [2] and intensification of a serine protease production at low temperatures [3].

On the other hand, fluorescence is a phenomenon of light emission by an excited molecule or atom, most often in the UV range, while light is emitted in the visible spectrum and takes on a specific wavelength. Siderophores are compounds secreted by bacteria and filamentous fungi to chelate iron ions, with which they form water-soluble complexes. This process increases the concentration of iron forms, available to organisms in the environment. It was confirmed that these compounds are also capable of binding Mn^2+^, Cr^3+^, Cd^2+^, Cu^2+^ or Pb^2+^ ions [4]. Siderophores can be divided due to their chemical structure—hydroxamic, ketocholic and mixed—or because of their origin—bacterial, fungal and components. The green-yellow fluorescent pigment secreted by *Pseudomonas* has been studied since 1978 [5] and has been designated as a siderophore-piperidin. It is considered to be characteristic for fluorescent strains and it was confirmed that the structure of the protein chain is significant when recognizing a siderophore through cellular receptors. It is specific for individual bacterial strains and is responsible for strong iron-binding capacity [6,7]. Due to the ability to chelate iron ions, the bacteria that produce siderophores are considered as growth stimulants for plants and as biopesticides. It is possible to limit the growth and development of phytopathogens by bacteria that occupy an ecological niche with a reduced pool of available iron. The pioverin-secreting bacteria succeed in competition by colonization of the rhizosphere and reduction in fungal growth. The second important path of research on siderophores is their application in medicine. The last few years have been a period of intense research on the so-called tactics of the Trojan horse. It is a method that allows for a selective inactivation of specific bacterial species. In this therapy, siderophore drug conjugates (SDCs) are used, i.e., a complex of a synthetic isophorus combined with an antibiotic and an iron ion. When the bacterial receptor system uptakes iron, it also receives a combined antibiotic, resulting in cell death. It is a system that gives opportunity to combat microbial resistance mechanisms—active drug removal and blocking hydrolysis [8].

Among various microorganisms isolated from the Arctic Ocean, psychrotrophic bacteria from the genus *Pseudomonas* represent the predominant group. *Pseumonas antarctica*, *P. brassicacearum* and *P. mandelii* belong to the *Pseudomonas fluorescens* group, while the taxonomy of *P. frederiksbergensis* is not yet fully established [9,10,11]. *Pseudomonas antarctica* bacteria are motile in the environment and are capable of growth at temperatures of between 4 and 30 °C, although their temperature optimum for growth is 22 °C. The bacteria tolerate an environment with 3% salinity. The optimal pH is 7.0. *P. antarctica* utilizes many carbon sources, including: d-galactose, d-glucose, glycerol, meso-inositol, d-mannitol, and produces catalase, oxidase, urease, phosphatase and moderately lipase. They do not hydrolyze esculin, starch and cellulose [12]. *Pseudomonas brassicacearum* is known mainly for colonizing root systems of *Arabidopsis*, cabbage or rapeseed. It produces a fluorescent pigment during growth on the CAA substrate [13,14]. *P. brassicacearum* oxidates such substrates as: *N*-acetylglucosamine, d- and l-alanine, l-arabinose, d-galactose, d-glucose, l-glutamic acid and d-mannose [15]. It also demonstrates the ability to suppress plant pathogens by producing antifungal compounds, e.g., cyanides [15]. *Pseudomonas mandela* bacteria, exhibits lower fluorescence on King’s B. Substrate showing catalase and oxidase positive capacities. Capable of growing at low temperatures at 4 °C, their temperature optimum is 30 °C. They thrive on low salinity <0.8% but are unable to grow in an environment with a salinity of 5% and 7% and at temperatures above 40 °C. Under anaerobic conditions, they are capable of hydrolysing arginine [16]. *Micrococcus luteus* is a very diverse group of cocci, especially in terms of cell wall structure. Differences mainly concern chemotaxonomy and are not found in other species of bacteria. Their common feature is the yellow color and spherical shape of the colony. All strains also utilize d-mannose, d-maltose and d-trehalose and casein as a carbon source [17].

The aim of this study was the isolation and identification of new bacteria from driftwood floating over the Arctic Ocean near Spitzbergen, followed by characterization of their enzymatic potential, in relation to their applicability in biodegradative processes, as well as the production of fluorescent dyes.

## 2. Results and Discussion

Based on the assessment of the morphology of colonies and microscopic evaluation, 117 pure cultures were obtained. Mass spectrometry of isolates using the MALDI technique resulted in the elimination of microorganisms with potential pathogenic characteristics and reduced the final number to 74 strains (Table 1).

From the bacterial isolation procedure on various media enriched with selected natural polymeric substrates the majority of isolates were successfully identified with both applied methods, MALDI-TOF Mass Spectrometry Biotyper and the analysis of 16S rDNA sequences. The MALDI-TOF Biotyper is a highly convenient and rapid method for the accurate identification of microorganisms, especially of clinical origin. The results from both methods are in general agreement, as to the assignment of the isolates to genera. In the case of 19% of the isolates, the two identification methods provided assignment to different genera; however, the same phyla were mostly in accordance, excluding cases where the proteomic method produced no result. It is noteworthy that MALDI-TOF MS Biotyper represents an emerging technique which relies on the quality of available databases, which, in turn, usually focus on clinical isolates. Hence, the availability of data for environmental species may be limited. On the contrary, the fact must be considered that sole analysis of 16S rDNA is considered insufficient in the case of some specific genera. The problem is especially vital, e.g., for bacterial genera *Pseudomonas* or bacilli from the *B. cereus* group. Some authors propose to include genes other than ribosomal. Mulet et al. [18] verified that *gyrB, rpoB* and *rpoD* genes were most useful for the multilocous sequence typing (MLST) identification technique of *Pseudomonas* sp. Nevertheless, in Mulet et al.’s [18] analysis of other gene sequences, i.a., *pycA, ccpA* or, *plcR, cerA*, or the 16S-23S rRNA ITS region were suggested as a supplementary factor for sufficiently distinguishing *B. cereus* from *B. thuringensis* and *B. anthracis* [19,20]. An even extended set of loci was proposed for the MLST of *Burkholderia* genus [21].

Predominant phyla obtained in the study comprised actinobacteria and gamma-proteobacteria, 40% and 37%, respectively. Firmicutes, mainly from the genus *Bacillus*, represented 10% of the isolates. The share of beta-proteobacteria and alpha-proteobacteria was 8% and 4%, respectively. Prevailing genera among actinobacteria were represented by *Arthrobacter* sp. (38%) and *Rhodococcus* sp. (34%). Bacteria from the genus *Pseudomonas* constituted 89% of the gamma-proteobacteria. The obtained results allow us to denote high similarity in MALDI-TOF MS Biotyper and PCR-based identification of bacterial isolates. According to different authors [22,23,24], the results were either similar or better in favor of MALDI-TOF MS Biotyper. 

**Figure 1:**
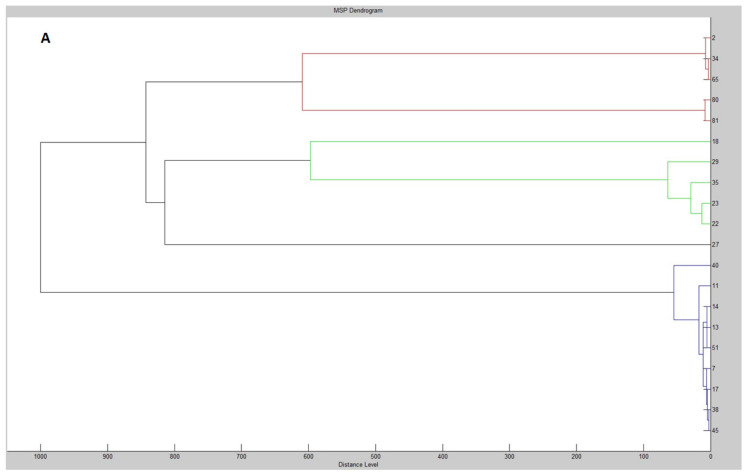

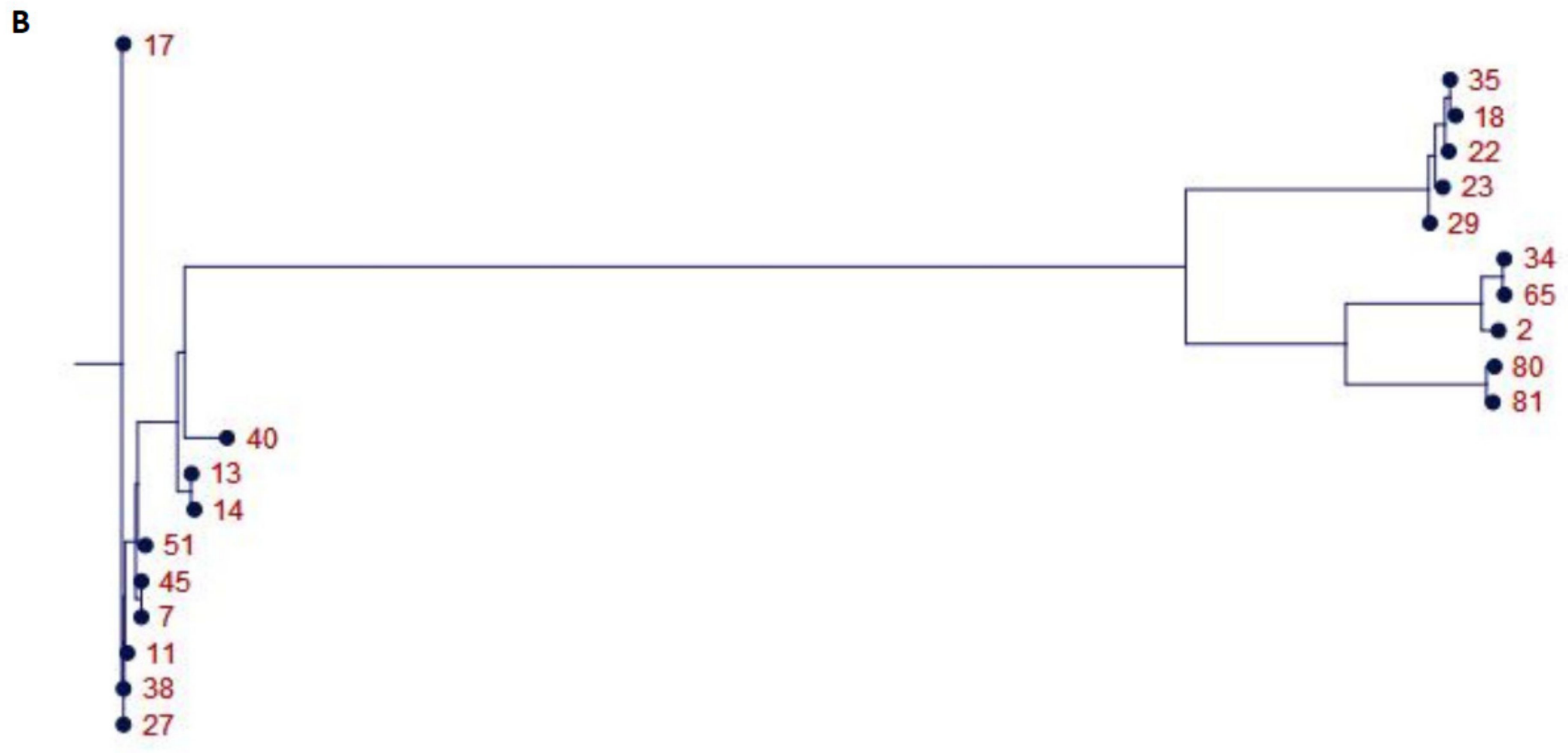
An example comparison of phylograms derived from MALI-TOF MS Biotyper (**A**) and 16S rDNA (**B**) analysis.

The phylogenetic analysis of both proteomic profiles and 16S rDNA sequences led to a conclusion that despite the identical assignment to species, phylogenetic relationships between isolates often vary. As an example, in the phylograms for both methods, the isolates *A. citreus* WG2, WG34 and WG65 belonged to the same cluster, as well as *M. luteus* WG80 and WG81 (Figure 1). On the contrary, the isolates WG29 and WG35 grouped within one cluster according to MALDI-TOF MS Biotyper did not reflect 100% similarity according to the 16S rDNA analysis. This is in accordance with the opinion of Strejcek et al. [25], who established that, although whole-cell MALDI-TOF-based identification of bacterial isolated is in general agreement with the results of 16S rRNA gene sequence analysis, high accordance was confirmed merely up to the genus level. Nevertheless, at the species level, the agreement appeared to be limited.

The analysis of hydrolytic potential of bacterial isolates allowed us to verify cellulolytic activity in 24% of the strains (over 0.05 U), while 10% of the isolates exhibited the activity exceeding 0.1U. The activity of xylanases and polygalacturonases was more common among the isolates, found in 25% and 22% of isolates, respectively, at levels higher than 0.1 U. Proteolytic microorganisms were sparse among the isolates, where merely 7% revealed activity over 0.05 U (Table 1). 

Arctic sea water represents an extreme environment that hosts a variety of bacterial species. According to the insight of Timperio et al. [24], the natural arctic water environment is dominated by gamma-proteobacteria, predominantly from the genus *Pseudomonas* and *Serratia*, that represent 76% of culturable microflora. The share of *Sphingobacteria* and *Actinobacteria* was 9% and 7%, respectively, while the occurrence of bacilli and flavobacteria did not exceed 4%. 

In our study, we used specific sites for the isolation of arctic microorganisms, which were the remains of wooden logs, where an obvious selection of microflora occurred towards the utilization of lingo-cellulosic material. Hence, the profile of isolated bacteria did not reflect the typical quality of marine microflora. In our study, the predominance of actinobacteria, followed by gamma-proteobacteria, was characteristic. 

Ferres et al. [26] conducted an isolation of arctic bacteria with special regard to their cellulolytic and lipolytic potential, from different sites, including water, icebergs, sediments and seaweed. Habitats rich in lingo-cellulosic material were a source of cellulase-producing microorganisms, which, on the contrary, were dominated by gamma-proteobacteria, including *Psychrobacter* sp. and *Pseudoalteromonas* sp. *Pseudomonas* sp. strains obtained by the authors dominated in the total number of isolated bacteria; however, they did not exhibit considerable cellulolytic and xylanolytic potential. 

**Figure 2:**
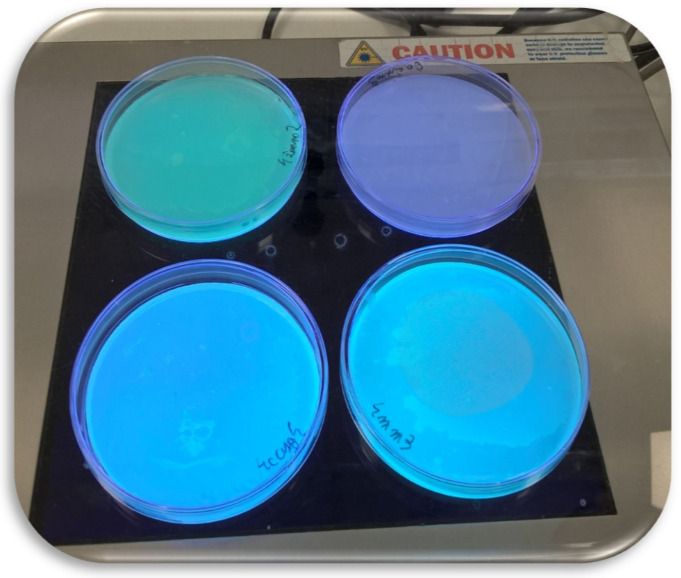
Example of fluorescence intensity of pigments produced by *Pseudomonas sp.* WG 7, WG 14 and WG 40, respectively, relative to control (purple plate), after 5 days of incubation.

Nine of the *Pseudomonas* sp. isolates exhibited the capability of fluorescent dye production. This feature was observed during growth in skim milk agar or pure nutrient broth medium (Figure 2). The fluorescence intensity of the pigment diffused into culture media, observed in UV light, varied among isolates. The highest fluorescence intensity was observed in the case of the *P. antarctica* WG40. 

**Figure 3:**
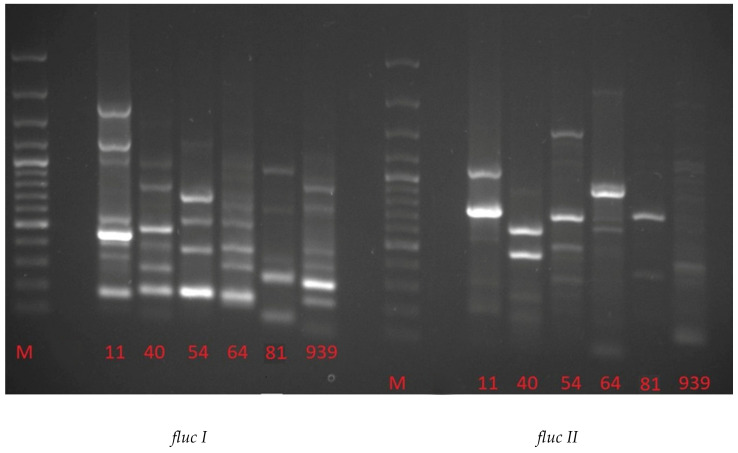
Electrophorogram obtained from PCR with fluc I and fluc II primers (M—marker GeneRuler 1000 kb).

Two pairs of universal primers, flucI and flucII, were used to amplify genes related to fluorescence [20]. The obtained excerpts are shown in the electrophorogram (Figure 3). PCR products amplified with the fluc I primer were obtained for *P. frederiksbergensis* 11,* P. antarctica* 40, *P. brassicacearum* 54, *P. mandelii* 64 and *P. aeruginosa* 939, where the product size ranged from 150 to 170 kb and from 300 to 380 kb. The only strain exhibiting fluorescence for which no product was detected was *Micrococcus luteus* 81. *P. antarctica* 40 as the only strain showed amplification product of 500–600 kb. 

In the case of *P. brassicacearum* 54, the only product exhibited 676 kb, and for *P. frederiksbergensis* 11 it was 1000 and 1647 kb. Two products of the same range of base pairs, from 400 to 500 kb, occurred in *P. frederiksbergensis* 11 and *P. antarctica* 40, but also 760–780 bp products in *P. antarctica* 40 and *P. aeruginosa* 939 and 1200–1250 kb products in *P. frederiksbergensis* 11 and *P. brassicacearum* 54. Amplification products between 930 and 995 kb occurred in 3 strains: *P. antarctica* 40, *Micrococcus luteus* 81 and *P. mandelii* 64. Four strains also have a product between 210 and 293 kb: *P. antarctica* 40, *P. mandelii* 64, *Micrococcus luteus* 81 and *P. aeruginosa* 939 (Table 2). Application of the fluc II primer resulted in fewer amplification products with a similar amount of pairs compared to fluc I. For the majority of strains, a product of 300–370 kb was observed, except for *P. antarctica* 40. *P. brassicacearum* 54 and *P. mandelii* 64 showed an amplification product of 488 kb. A smaller product of 434 kb was obtained in the case of the *P. antarctica* 40 strain. Products with a size 500–590 kb were observed in *P. frederiksbergensis* 11, *P. antarctica* 40, *P. mandelii* 64 and *P. aeruginosa* 939, indicating that the product of the last two was similar (Table 2, Figure 3). 

According to Meyer and Stintzi [27], approximately 30 genes are related to the bacterial siderophores synthesis and are responsible for the production of both pioverdin and piochielin. The fusion system of *P. aeruginosa* ATCC 15692 was thoroughly described, for which the biosynthesis genes have been mapped. The pvdA gene of 426 bp has been tagged as responsible for the production of mRNA to synthesize the enzyme l-ornithine *N^5^*-oxygenase [28]. It is a gene widely distributed among the *Pseudomonas* genus. In our study, the PCR amplification products in with similar number of pairs have been obtained for *Pseudomonas* strains, in addition to *P. aeruginosa* 939 and M. luteus 81. The gene for the likely features of oxygenases (pvcB) and gene transfer in cytochrome C (pvcD) located by Meyer and Stintzi [27] and have sizes of 289 and 215 amino acid residues, respectively. All strains also showed the amplification product of 500–600 kb, a gene potentially associated with hydroxylases. The product of approximately 500 kb was characteristic for *P. mandelii* 64 and *P. brassicacearum* 54, the larger product of approximately 589 kb—for *P. antarctica* 40, *P. mandelii* 64, *M. luteus* 81 and *P. aeruginosa* 939.

## 3. Materials and Methods

The evaluation of the enzymatic potential of microorganisms isolated from wooden logs adrift in the Spitzbergen area was performed. Fragments of surface material of approximately 5 mm depth were aseptically removed. Samples of 5 g were collected and stored refrigerated in sterile falcon tubes. Subsequently, entire samples were placed in 100 mL of sterile 0.85% saline and agitated for 30 min on a rotary shaker (VWR, 160 rpm). Isolation, using 0.1 mL of the suspension, by a classical plate method on agar plate was carried out in order to gain isolates capable of growth and utilization of specific natural polymers as a source of carbon. For this purpose, 1% solutions/suspensions of the following substrates were used: colloidal chitin (Sigma-Aldrich, Poznań, Poland) (towards chitinases), laminarin (Sigma-Aldrich) (towards glucanases), cellulose and carboxymethylcellulose (Sigma-Aldrich) (towards cellulases), xylan (Sigma-Aldrich) (towards xylanases), casein from bovine milk (Sigma-Aldrich) (towards proteases), tributyrin (Sigma-Aldrich) and vegetable oils (olive oil, food grade) (towards lipases). The incubation of the plates was conducted at 30 and 4 °C. 

MALDI-TOF MS Biotyper (Bruker Daltonik, Billerica, MA, USA) isolation and purification of microbial cultures was performed by plate method to obtain pure cultures. Biomass from a single colony was suspended in sterile distilled water (300 μL) and supplemented with absolute alcohol (900 μL). After centrifugation, 20 μL formic acid was added and the homogenization of cells was performed, followed by the addition of 20 μL acetonitrile solution to sediment. After centrifugation, 1 μL of the supernatant was applied on the plate. After drying, 1 μL of cyano-4-hydroxycinanic acid (HCCA) was added [29]. Determinations were performed at 2–10 kDa m.w. range, linear positive mode, ion source I: 20 kV, ion source II: 19 kV, lens voltage 6.5 kV. 

For the selected group of bacteria, molecular analysis of species affiliation was performed, preceded by 24 h cultures for DNA isolation using the Genomic Mini kit, Genomic Mini AX Yeasts SPIN and PCR master mix (according to manufacturer’s protocol), and PCR reaction with universal 16S rDNA primers 27F (AGAGTTTGATCGTGGCTCAG) and 1491R (GGTTACCTTGTTACGACT) [30]. The obtained products were subjected to sequencing (GENOMED S.A., Polska—sequencing reactions were performed using the BigDye Terminator v3.1 kit from Applied Biosystems (Thermo Fisher Scientific Inc., Waltham, MA, USA). The sequencing reaction products were separated on a 3730x1 DNA analyzer capillary sequencer, Thermo Fisher Scientific Inc., Waltham, MA, USA) and compared to the Ribosomal Database Project (RDP) Release 11. A phylogenetic analysis was performed using the CLC Sequence Viewer 7 tool (QIAGEN, Venlo, Netherlands).

The evaluation of the enzymatic potential was carried out in agitated flask cultures in 25 mL of media, composed of: peptone 0.5%, glucose 0.2%, sugar beep pulp 1%. The incubation time was 3 days at room temperature.

Enzymatic activities, according to released free sugars from individual polymers, were determined using a DNS (3,5-dinitrosalicilic acid) reagent [31].

Fluorescence levels were evaluated visually in UV radiation as well as by the analysis of PCR products of fluorescence-related genes. In order to identify genome fragments responsible for the production fluorescent substances, PCR was performed. Biomass was obtained after a 3 day cultivation in a medium containing 3% skim milk and nutrient broth. The genetic material was isolated using “Genomic Mini Kit—a kit for the isolation of genomic DNA from bacteria in culture Cells and Solid Tissue” (A&A Biotechnology). The universal primers (fluc I (f) GCCTCCCTCGCGCCA/(r) GCCTTGCCAGCCCGC; fluc II (f) CAGGACCAGGCTACCGTG/(r) CGGAGAGCCGAGAGGTG ) used in the study are capable of amplifying multiple loci and are highly amplified in the early cycles of PCR. The PCR reaction mixtures contained 25 μL Taq PCR Master Mix (2x) (Eurx), 20 pmol of each primer, and 10 μL genomic DNA. The PCR was carried out with initial denaturation of 94 °C for 5 min, followed by 35 cycles of denaturation at 94 °C for 1 min, annealing at 60 °C (flucI); 59 °C (flucII) for 30 s, extension at 72 °C for 90 s and a final extension at 72 °C for 10 min [32].

## 4. Conclusions

We have obtained 74 isolates from a specific niche in the form of driftwood from the Arctic region. The strains identification based on the analysis of 16S rDNA and MALDI-TOF MS Biotyper, allowed for their assignment to genera or species, with general agreement between the two methods. The pool of isolates was dominated by actinobacteria and gamma-proteobacteria; however, the composition of microflora did not reflect typical marine microflora. The inquiry towards hydrolytic activities revealed prevalent xylanolytic capabilities among the tested microorganisms. Additionally, ten fluorescent dye-producing isolates, mainly of *Pseudomonas* genus, were determined. The most intense fluorescence was demonstrated by the *Pseudomonas antarctica* 40 strain cultivated on skim milk medium, and with milk and casein. The specific collection of Arctic origin bacteria with confirmed hydrolytic capabilities could be useful in biotechnological processes aimed at the biodegradation of lignocellulosic materials. Nevertheless, further optimization of the culture conditions for specific enzyme production or exact substrate breakdown is required.

## Figures and Tables

**Table 1:** Identification results of isolates with corresponding enzymatic activities (maximum values given, U/mL) and occurrence of fluorescence.

	MALDI-TOF MS Biotyper	16S rDNA	CEL	XYL	PG	PROT	FL
WG 1	*Arthrobacter oryzae*	*Arthrobacter oryzae*	0	0.18	0.06	0	
WG 2	*Arthobacter citreus*	*Arthrobacter citreus*	0.05	0.49	0.12	0	
WG 3	*Arthrobacter* sp.	*Arthrobacter* sp.	0.03	0	0.12	0	
WG 4	*Bacillus cereus*	*Bacillus* sp.	0	0	0	0	
WG 5	*Pseudomonas* sp.	*Pseudomonas* sp.	0.06	0.18	0	0.01	
WG 7	*Pseudomonas frederiksbergensis*	*Pseudomonas* sp.	0	0.02	0.07	0.02	+
WG 8	*Bacillus weihenstephanensis/mycoides*	*Bacillus mycoides*	0	0	0	0	+
WG 9	*Pseudomonas frederiksbergensis*	*Pseudomonas frederiksbergensis*	0.03	0	0	0	
WG 11	*Pseudomonas frederiksbergensis*	*Pseudomonas frederiksbergensis*	0	0	0	0.01	
WG 12	*Mycobacterium* sp.	*Massilia* sp.	0.15	0.20	0.04	0	
WG 13	*Pseudomonas frederiksbergensis*	*Pseudomonas* sp.	0.10	0.59	0.31	0	
WG 14	*Pseudomonas frederiksbergensis*	*Pseudomonas* sp.	0	0	0	0	+
WG 15	*Rhodococcus erythopolis*	*Rhodococcus erythropolis*	0	0.04	0	0	
WG 16	*Pseudomonas* sp.	*Pseudomonas frederiksbergensis*	0.11	0.39	0.17	0	
WG 17	*Pseudomonas frederiksbergensis*	*Pseudomonas frederiksbergensis*	0.19	0	0	0.06	
WG 18	*Rhodococcus* sp.	*Rhodococcus erythropolis*	0	0	0.09	0	
WG 19	*Rhodococcus erythopolis*	*Rhodococcus erythopolis*					
WG 20	*Rhodococcus erythopolis*	*Rhodococcus erythropolis*	0	0.01	0.06	0.03	
WG 21	*Pseudomonas* sp.	*Pseudomonas* sp.	0	0.05	0	0	
WG 22	*Rhodococcus erythopolis*	*Rhodococcus erythropolis*	0	0	0	0	
WG 23	*Rhodococcus erythopolis*	*Rhodococcus erythropolis*	0	0	0	0	
WG 25	*Pseudomonas* sp.	*Pseudomonas* sp.	0.06	0.42	0	0	
WG 26	*Arthobacter citreus*	*Arthobacter citreus*	0	0.05	0.07	0.01	
WG 27	*Rhodococcus erythopolis*	*Pseudomonas frederiksbergensis*	0	0.42	0.44	0	
WG 28	*Acinetobacter* sp.	*Acinetobacter* sp.	0.08	0	0.01	0	
WG 29	*Rhodococcus erythopolis*	*Rhodococcus erythropolis*	0.02	0.04	0.01	0.05	
WG 30	*Rhodococcus erythopolis*	*Rhodococcus erythopolis*					
WG 34	*Arthobacter citreus*	*Arthrobacter citreus*	0.02	0.03	0.05	0.02	
WG 35	*Rhodococcus erythopolis*	*Rhodococcus erythropolis*	0.01	0	0.01	0.02	
WG 36	*Bacillus* sp.	*Sporosarcina aquimarina*	0	0.26	0.11	0.08	
WG 38	*Pseudomonas* sp.	*Pseudomonas frederiksbergensis*	0	0	0.05	0	+
WG 40	*Pseudomonas antarctica*	*Pseudomonas antarctica*	0.02	0.01	0.05	0	+
WG 43		*Pseudomonas arsenicoxydans*	0	0	0.20	0	
WG 44	*Pseudomonas frederiksbergensis*	*Pseudomonas frederiksbergensis*	0.08	0	0	0.12	+
WG 45	*Pseudomonas frederiksbergensis*	*Pseudomonas frederiksbergensis*	0	0.77	0	0.01	
WG 46		*Sphingomonas* sp.	0	0.3	0	0	
WG 47	*Rhodococcus erythopolis*	*Rhodococcus erythopolis*	0	0.07	0.03	0	
WG 51	*Pseudomonas* sp.	*Pseudomonas* sp.	0.09	0.08	0.30	0	+
WG 53	*Pseudomonas frederiksbergensis*	*Pseudomonas frederiksbergensis*	0	0.01	0.10	0	
WG 54	*Pseudomonas brassicacearum*	*Pseudomonas brassicacearum*	0	0.01	0.01	0	
WG 56	*Pseudomonas koreensis*	*Pseudomonas koreensis*	0.01	0	0.09	0	
WG 60	*Pseudomonas* sp.	*Pseudomonas* sp.	0.02	0.05	0.18	0.02	
WG 61	*Pseudomonas frederiksbergensis*	*Pseudomonas frederiksbergensis*	0	0	0	0	+/−
WG 62	*Pseudomonas corrugata*	*Pseudomonas corrugata*	0	0.04	0.09	0	
WG 64	*Pseudomonas mandelii*	*Pseudomonas mandelii*	0	0.02	0	0.01	+
WG 65	*Arthobacter citreus*	*Arthrobacter citreus*	0.20	0	0.09	0	
WG 67		*Burkholderia sordidicola*	0.04	0	0.28	0	
WG 69	*Bacillus psychrosaccharolyticus*	*Bacillus psychrosaccharolyticus*	0	0	0	0.01	
WG 71	*Pseudomonas* sp.	*Pseudomonas* sp.	0	0.03	0.58	0	
WG 72		*Burkholderia* sp.	0.16	0	0	0.05	
WG 76	*Lactobacillus* sp.	*Lactobacillus* sp.	0.011	0.39	0.04	0	
WG 77	*Micrococcus luteus*	*Micrococcus luteus*	0.09	0.01	0.06	0	
WG 79		*Arthrobacter oxydans*	0.22	0.47	0	0	
WG 80	*Micrococcus luteus*	*Micrococcus luteus*	0	0.13	0	0	
WG 81	*Micrococcus luteus*	*Micrococcus luteus*	0	0.92	0	0	+
WG 82	*Micrococcus luteus*	*Micrococcus luteus*	0	0	1.60	0	
WG 83	*Micrococcus luteus*	*Micrococcus luteus*	0.02	0.03	0.33	0.01	
WG 85		*Arthrobacter* sp.	0.07	0	0.03	0	
WG 89	*Arthobacter polychromogenes*	*Burkholderia* sp.	0.01	0.16	0.17	0	
WG 93		*Burkholderia* sp.	0	0	0.77	0	
WG 94	*Aerococcus vividans*	*Sphingomonas* sp.	0.03	0	0.03	0	
WG 95		*Sphingomonas* sp.	0.01	0	0.05	0	
WG 99	*Cupriavidus metallidurans*	*Sphingomonas* sp.	0.05	0	0.28	0.01	
WG 100	*Arthobacter sufonivorans*	*Arthobacter sufonivorans*	0	0.2	0.03	0	
WG 101	*Cupriavidus metallidurans*	*Sphingomonas* sp.	0	0.05	0.07	0	
WG 103	*Mycobacterium sp.*	*Caulobacter* sp.	0	0.9	0	0.02	
WG 104	*Arthobacter* sp.	*Arthobacter* sp.	0	0	0.03	0.05	
WG 108	*Arthobacter ramosus*	*Arthrobacter* sp.	0	0	0.05	0	
WG 113	*Mycobacterium* sp.	*Mycobacterium* sp.	0	0	0	0	
WG 114	*Bacillus simplex*	*Bacillus simplex*	0.08	0	0	0	
WG 116	*Acinetobacter baumanii*	*Frondihabitans* sp.	0.01	0.02	0.05	0.01	
WG 117	*Paenibacillus pabuli*	*Streptococcus salivarius*	0.01	0.04	0.03	0.01	

CEL—cellulolytic activity, XYL—xylanolytic activity, PG—polygalacturonase activity, PROT—proteolytic activity, FL—fluorescent dye production.

**Table 2:** Base pair length of amplification products using fluc I and fluc II primers.

Fluc I						Fluc II					
STRAINS						STRAINS					
11	40	54	64	81	939	11	40	54	64	81	939
									2262		2045
1647								1516			
1207		1244									
1000						1047		1192			1131
	988		994	934							
							889		856		805
	771				767	729					
		676						682	668	691	
517		500	593	586	593	520	582		589		589
			500				434	488	488		
438	461					303		310	303	339	362
308	376	367	376		344	202	245				
	270		280	292	275		187		74		96
				232	216						
174	179	167	155	80	147						
